# Habit training versus habit training with direct visual biofeedback in adults with chronic constipation: study protocol for a randomised controlled trial

**DOI:** 10.1186/s13063-017-1880-0

**Published:** 2017-03-24

**Authors:** Christine Norton, Anton Emmanuel, Natasha Stevens, S. Mark Scott, Ugo Grossi, Sybil Bannister, Sandra Eldridge, James M. Mason, Charles H. Knowles

**Affiliations:** 1 King’s College, 57 Waterloo Road, London, SE1 8WA UK; 2 University College Hospital, 235 Euston Road, London, NW1 2BU UK; 3 Blizard Institute, Queen Mary University of London, 2 Newark Street, London, E1 2AT UK; 4 Blizard Institute, Queen Mary University of London, 4 Newark Street, London, E1 2AT UK; 5 Warwick Medical School, University of Warwick, Coventry, CV4 7AL UK

**Keywords:** Constipation, Biofeedback, Behavioural intervention, Randomised controlled trial, Anorectal testing

## Abstract

**Background:**

Constipation affects up to 20% of adults. Chronic constipation (CC) affects 1–2% of adults. Patient dissatisfaction is high; nearly 80% feel that laxative therapy is unsatisfactory and symptoms have significant impact on quality of life. There is uncertainty about the value of specialist investigations and whether equipment-intensive therapies using biofeedback confer additional benefit when compared with specialist conservative advice.

**Methods/design:**

A three-arm, parallel-group, multicentre randomised controlled trial.

Objectives: to determine whether standardised specialist-led habit training plus pelvic floor retraining using computerised biofeedback is more clinically effective than standardised specialist-led habit training alone; to determine whether outcomes are improved by stratification based on prior investigation of anorectal and colonic pathophysiology. Primary outcome measure is response to treatment, defined as a 0.4-point (10% of scale) or greater reduction in Patient Assessment of Constipation–Quality of Life (PAC-QOL) score 6 months after the end of treatment. Other outcomes up to 12 months include symptoms, quality of life, health economics, psychological health and qualitative experience.

Hypotheses: (1) habit training (HT) with computer-assisted direct visual biofeedback (HTBF) results in an average reduction in PAC-QOL score of 0.4 points at 6 months compared to HT alone in unselected adults with CC, (2) stratification to either HT or HTBF informed by pathophysiological investigation (INVEST) results in an average 0.4-point reduction in PAC-QOL score at 6 months compared with treatment not directed by investigations (No-INVEST).

Inclusion: chronic constipation in adults (aged 18–70 years) defined by self-reported symptom duration of more than 6 months; failure of previous laxatives or prokinetics and diet and lifestyle modifications. Consenting participants (*n* = 394) will be randomised to one of three arms in an allocation ratio of 3:3:2: [1] habit training, [2] habit training and biofeedback or [3] investigation-led allocation to one of these arms. Analysis will be on an intention-to-treat basis.

**Discussion:**

This trial has the potential to answer some of the major outstanding questions in the management of chronic constipation, including whether costly invasive tests are warranted and whether computer-assisted direct visual biofeedback confers additional benefit to well-managed specialist advice alone.

**Trial registration:**

International Standard Randomised Controlled Trial Number: ISRCTN11791740. Registered on 16 July 2015.

**Electronic supplementary material:**

The online version of this article (doi:10.1186/s13063-017-1880-0) contains supplementary material, which is available to authorized users.

## Background

Constipation is common in adults and children and up to 20% of the population report this symptom depending on definitions used (2–28% adults; 0.7–30% children) [1–3], with a higher prevalence in women [1, 4, 5] and older people [6, 7]. Chronic constipation (CC), usually defined as more than 6 months of symptoms, is less common [8] but results in 0.5 million UK GP consultations per annum. A proportion of the population suffer symptoms which are both chronic and disabling (approximately 1–2% of the population) [9]. Such patients, who are very frequently female [10], are usually referred to secondary care with many progressing to tertiary specialist investigation. Patient dissatisfaction is high in this group; nearly 80% feel that laxative therapy is unsatisfactory [11] and the effect of symptoms on quality of life (QOL) is significant [12]. Constipation consumes significant health care resources. In the US in 2012, a primary complaint of constipation was responsible for 3.2 million physician visits [13] resulting in (direct and indirect) costs of US$1.7 billion. In the UK, it is estimated 10% of district nursing time is spent on constipation [14] and the annual spend on laxatives exceeds £80 million, with 17.4 million medication prescriptions in 2012 [15].

The act of defaecation is dependent on the coordinated functions of the colon, rectum and anus. Considering the complexity of neuromuscular (sensory and motor) functions required to achieve planned, conscious and effective defaecation [16] it is no surprise that disturbances to perceived ‘normal’ function occur commonly at all stages of life. Clinically, such problems often lead to symptoms of obstructed defaecation, e.g. straining; incomplete, unsuccessful or painful evacuation; bowel infrequency; abdominal pain and bloating. After exclusion of a multitude of possible secondary causes (obstructing colonic lesions, neurological, metabolic and endocrine disorders), the pathophysiology of CC can broadly be divided into problems of colonic contractile activity (and hence stool transit) and problems of evacuation and the pelvic floor. Thus, with specialist physiological investigation (hereafter referred to as INVEST in this protocol), patients may be divided into those who have slow colonic transit, evacuation disorder, both or neither (no abnormality found with tests). Evacuation disorders can be then subdivided into those in whom a structurally significant pelvic floor abnormality is evident, e.g. rectocoele or internal prolapse (intussusception) and those in whom there is a dynamic failure of evacuation without structural abnormality: most commonly termed ‘functional defaecation disorder’ (FDD).

Management of CC is a major problem due to its high prevalence and lack of widespread specialist expertise. In general, a step-wise approach is undertaken, with first-line conservative treatment, such as lifestyle advice and laxatives (primary care), followed by conservative bowel retraining programmes, sometimes including focussed biofeedback and psychosocial support (secondary/tertiary care). Although these treatments may improve symptoms in more than half of patients, they are very poorly standardised and may not improve the whole range of patient symptoms. The care pathway for nonresponders then separates into two main routes, determined by local availability of care and clinician preference: some patients are left to self-manage often very intrusive symptoms, while others are offered a range of costly, irreversible surgical interventions with unpredictable long-term results [17, 18], sometimes resulting in major adverse events (AEs) or a permanent stoma.

The current trial forms part of a UK National Institute of Health Research-funded programme grant (PGfAR: RP-PG-0612-20001: CapaCiTY). This programme aims to develop the evidence base for the management of CC in adults which is currently lacking. This is in contrast to the management of CC in children for whom UK national guidance has been published [19, 20]; and for adults with faecal incontinence [21]. Thus, the current situation is one where there are considerable variations in practice, particularly in specialist services. With a number of new drugs gaining or seeking UK National Health Service approval [22–25], and potential new technologies on the horizon [17, 26–28], it is timely that the currently limited evidence base is developed to provide confidence that new and sometimes expensive investigations and therapies are appropriate and cost-effective. A cost-conscious pathway of care may help to reduce health care expenditure by appropriately sequencing the care provided, while targeting more expensive therapies at those most likely to benefit. Such data will inform the development and commissioning of integrated care pathways.

The CapaCiTY research programme includes a series of interlinked studies that answer the important questions for patient care. A rolling programme of national recruitment will provide a large cohort of well-defined patients for three studies over 5 years (the present study plus studies of rectal irrigation and surgery for CC). The focus will be on generating real-life evidence from pragmatic studies which will provide valid clinical outcome measures, and address patient acceptability and cost. Armed with such data it will be possible to develop a management algorithm for CC which will meet patient, clinician and policy aims.

### Rationale for choice of comparators

In most UK practices, patients are first referred to a colorectal specialist for a variety of behavioural interventions to improve defaecatory function. A range of cohort studies [29], randomised controlled trials (RCTs) [30–35], reviews [36], guidelines [37], meta-analysis [38] and a Cochrane review [39] attest to the general success of this approach. However, opinion varies greatly concerning the complexity of intervention required, which patients are suitable for which interventions, and UK survey evidence (unpublished data from our trial development work) indicates that there is remarkable variability of practice.

The most basic form of behavioural therapy comprises ‘habit training’ (HT). This involves optimising eating patterns to maximise the gastro-colic response which is associated with morning clustering of high-amplitude propagated colonic contractions which propel contents towards the rectum for subsequent evacuation [40]. Dietary advice to optimise intake of liquid and modifying intake of wheat, fibre and other elements that the individual has found helpful/unhelpful is given, as well as advice about frequency and length of toilet visits, posture and how to evacuate effectively without straining. Patients are also instructed on basic gut anatomy and function, and gain an appreciation of how psychological and social stresses may influence gut functioning. Simple pelvic floor exercises are often included.

More complex forms of therapy include instrument-based biofeedback learning techniques [29–35]. Favoured in the US, and by about half of UK centres, these provide direct visual computer-based biofeedback of pelvic floor activity, usually displayed as real-time pressure or electromyogram (EMG) activity during defaecation manoeuvres (e.g. ‘bearing down’) or attempted rectal balloon expulsion. It is noteworthy that most instrument-based programmes have a one to three sessions/week approach as opposed to the HT programmes which usually entail less frequent contact to allow behavioural change to take root. While small RCTs suggest an additive value of biofeedback over HT alone in the management of selected patient subgroups of CC [31, 41–43], there has been no multicentre or adequately powered RCT in unselected patients despite uncertain benefit and significant resource implications. There is even controversy about which subgroups benefit: some studies suggest that only those with proven puborectalis incoordination and no slow transit benefit from biofeedback [41, 42] while others are less exclusive [29, 31]. Further, most publications advocating biofeedback have come from specialist centres with considerable ‘investment’ in these techniques with much less favourable reports when biofeedback is the comparator in a trial of more invasive treatment [44, 45]. These data (and their heterogeneity) have been described in a recent Cochrane review [39].

Despite being widely employed, there is conflicting evidence as to whether radiological and physiological investigations influence outcomes in CC, with significant differences of expert opinion. Some advocate early complex and expensive investigations to guide treatment in most patients [9] whereas others undertake such tests only in resistant cases or those considering progressing to surgery [46]. The potential advantage of guiding treatment [42, 47] is balanced against the invasive nature of some tests, radiation exposure, embarrassment and cost (circa £600–1200 NHS tariff): all currently require an escalation of care from primary care to hospital, and most currently necessitate an escalation of care from a secondary to a tertiary centre. In addition it is not certain that results are improved by extensive investigation. The need to resolve this question has been consistently highlighted [37, 48, 49]. However, it can only be addressed satisfactorily by evaluating outcomes from treatment with or without these tests. Of particular relevance is the possibility that specialist-led therapies (as above) could be stratified using these tests. Notably, there is some evidence that HT with computer-assisted direct visual biofeedback (HTBF) may maximally benefit patients with certain pathophysiologies [35, 41–43, 50, 51], especially FDD [37, 52, 53].

**Fig. 1:**
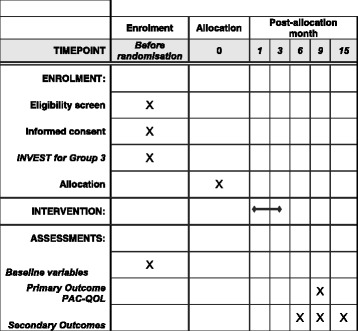
Standard Protocol Items: Recommendations for Interventional Trials (SPIRIT) figure

This protocol has been prepared in accordance with the Standard Protocol Items: Recommendations for Interventional Trials (SPIRIT) Checklist and figure (Additional file 1 and Fig. 1).

## Methods/design

### Primary objectives

1. To determine whether standardised specialist-led HT plus pelvic floor retraining using computer-assisted direct visual biofeedback (HTBF) is more clinically effective than standardised specialist-led habit training alone (HT)
2. To determine whether outcomes of such specialist-led interventions are improved by stratification to HTBF or HT based on prior knowledge of anorectal and colonic pathophysiology using standardised radio-physiological investigations (INVEST)

### Secondary objectives

1. To determine the cost-effectiveness of both interventions and INVEST
2. To qualitatively evaluate patient and health professional experience of interventions and INVEST

### Trial design

**Fig. 2:**
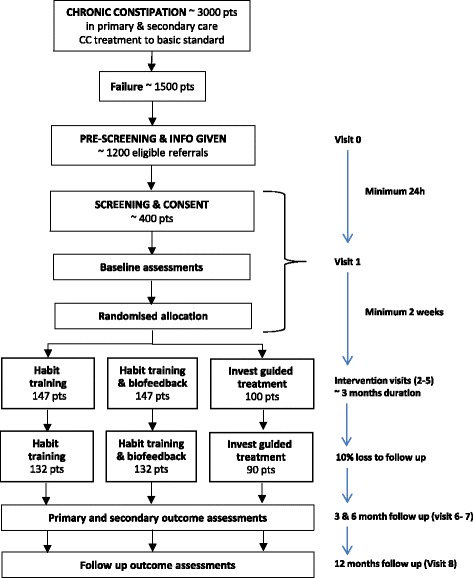
Study design

The study design is a three-arm, parallel-group, multicentre RCT. The trial provides two comparisons: HT (group 1) compared with HTBF (group 2); and investigation-led treatment (group 3) compared with management without investigation (groups 1 and 2) (Fig. 2). Based on our knowledge of the characteristics of the population we expect approximately 50% of those in group 3 to be allocated to each of the two treatments to which individuals are randomised in groups 1 and 2.

### Study setting

This trial will be conducted in 10 to 15 UK centres. Patients attending medical services (primary care through to specialist centres: outpatient clinics; gastrointestinal (GI) physiology units) for constipation will be eligible for recruitment and assessed against the eligibility criteria.

### Characteristics of participants

#### Eligibility criteria

##### Inclusion criteria

- Age 18–70 years;
- Patient self-reports problematic constipation
- Symptom onset more than 6 months prior to recruitment
- Symptoms meet American College of Gastroenterology definition [54] of constipation: ‘unsatisfactory defaecation characterised by infrequent stool, difficult stool passage or both for at least previous 3 months’
- Constipation failed treatment to a minimum basic standard (NHS Map of Medicine 2012 [55] (lifestyle *and* dietary measures *and* at least two laxatives or prokinetics tried – no time requirement)
- Ability to understand written and spoken English (due to questionnaire validity)
- Ability and willingness to give informed consent

##### Exclusion criteria

The major causes of secondary constipation and factors precluding participation in study interventions:

- Significant organic colonic disease (‘red flag’ symptoms, e.g. rectal bleeding prior investigated); inflammatory bowel disease; megacolon or megarectum (if diagnosed beforehand); severe diverticulosis, bowel stricture or birth defects deemed to contribute to symptoms (incidental diverticulosis if known is not an exclusion)
- Major colorectal resection surgery
- Current overt pelvic organ prolapse (bladder, uterus, rectum) or disease requiring obvious surgical intervention
- Previous pelvic floor surgery to address defaecatory problems: posterior vaginal repair, stapled rectal resection (STARR) and rectopexy; previous sacral nerve stimulation
- Rectal impaction (as defined by digital and abdominal examination: these form part of the NHS Map of Medicine basic standard) [55]
- Significant neurological disease deemed to be causative of constipation, e.g. Parkinson’s disease, spinal injury, multiple sclerosis, diabetic neuropathy (not uncomplicated diabetes alone)
- Significant connective tissue disease: scleroderma, systemic sclerosis and systemic lupus erythematosus (SLE) (not hypermobility alone)
- Significant medical comorbidities and activity of daily living impairment (based on the Bartel Index in apparently frail patients [56, 57]; Barthel Index ≤11)
- Major active psychiatric diagnosis (schizophrenia, major depressive illness and mania)
- Chronic regular opioid use (at least once daily use) where this is deemed to be the cause of constipation based on temporal association of symptoms with onset of therapy; all regular strong opioid use
- Previous specialist nurse or therapist-led bowel management
- Severe visual impairment sufficient to prevent visual biofeedback
- Pregnancy or intention to become pregnant during study period

### Study interventions

Note: all therapists attend a 1-day formal training event prior to commencing the trial. As part of intervention fidelity work we are also observing at least one session per therapist. We have detailed documents with Standard Operating Procedures (SOPs) for both the HT and HTBF sessions with checklists for therapists to complete at the end of each session so that we can check what has been covered with each participant. These SOPs are available from author NS on request. These are all therapists who are delivering biofeedback in routine clinical practice and in this trial they will all deliver both interventions.

#### Group 1: habit training (HT)

Habit training will be provided by trained specialists (e.g. nurses or physiotherapists) with clinical experience, who have undertaken an additional study-specific standard 1-day training session. A standardised approach and intervention will be provided via use of an intervention manual; at least one random observation visit will be performed early in the study by lead research nurses for quality control.

The course of therapy will include a minimum of three, maximum of four sessions (45–60 min each session). This will use a standardised session proforma with an interval tolerance of every 3–5 weeks. The first and last session will always be face to face. Sessions will be delivered by the same therapist if at all possible and tailored to participants’ individual needs. Each recruiting site will provide sufficient resource to cover visits in therapist absence or holiday. Participants will receive the interventions in Table 1 which are covered in a booklet given to each participant. The therapist is prohibited from using digital rectal examination to train manoeuvres and will complete relevant sections in the participant booklet advising on individual instructions.

**Table 1:** Interventions for habit training (HT – group 1)

a. Provision of a written information leaflet covering normal bowel function; causes of constipation; diet and fluid advice; getting into a good bowel habit
b. Review of written information using locally available teaching tools such as models or diagrams
c. Advice to stop all laxatives including drugs which have a laxative effect or over the counter herbal teas that contain strong purgatives. One or two glycerin suppositories are permitted as rescue if no stool is passed for 3 days. No use of irrigation devices or enemas
d. Encouragement to follow a daily routine: sit on the toilet for 20–30 min after first meal and/or hot drinks (sooner if urge felt)
e. Advice to attempt defaecation after meals or when urge is felt, but no more than 3 times per day
f. Advice to sit on toilet with knees bent to 45° position with feet elevated on stool or equivalent; abdominal brace and breathe while performing anal relaxation
g. Advice only to attempt to push for 5–10 min maximum
h. Teaching on defaecation manoeuvres, taught while the patient is positioned sitting on chair with verbal coaching to breathe while pushing
i. Strong discouragement from multiple attempts and prolonged straining
j. Advice not to digitate anally
k. Where appropriate, the participant will be taught rectocoele (vaginal), perineal and perianal splinting;
l. Therapist prohibited from using digital rectal exam to train manoeuvres
m. Diet and lifestyle advice, e.g. moderate but not excessive fibre; moderate but not excessive fluid intake; increase physical exercise, e.g. walking if possible
n. Participants with evacuation difficulty and/or perineal descent will be taught pelvic floor exercises
o. Plenty of optimism, encouragement and personal attention
p. Suggestions of what to work on until next intervention session
q. Therapist to complete relevant sections of patient booklet

#### Group 2: habit training plus computer-assisted direct visual biofeedback (HTBF)

Each session will incorporate all features of the HT intervention (Table 1), but also include direct visual biofeedback using a portable high-resolution anal manometry (HRaM) system and catheter connected to a laptop computer with large monitor screen (Table 2). Calibration, validation and maintenance of the equipment will follow the built-in programme and training manuals provided and recorded on the system at each session. Training on the system will be given prior to sites commencing treatment. Both the patient and the therapist will be looking at the screen during the HTBF sessions. We are using state-of-the-art, high-resolution manometry equipment which displays a continuous colour display of rectal and anal pressures in real time. The patient can clearly see if attempted squeeze or attempted balloon expulsion changes anal or rectal pressures. The therapist can draw the patient’s attention to aspects of the display and coach the patient to improve performance. After each session the therapist will note the ability to expel the balloon, generate propulsion, increase rectal pressure, relax the anal canal, and the ability to sense the balloon at lower or higher volumes (relevant to hyposensate and hypersensate patients).

**Table 2:** Additional interventions at each session for group 2

a. Biofeedback balloon and catheter/probe will be connected to the manometry system and linked to the computer monitor. Patient lying in lateral position facing computer screen (supine if unable to lie in lateral position). Probe taped or held into position
b. Resting and squeeze pressure noted
c. Rectal balloon inflated with air at 2 mL/s to assess first sensation, urge sensation and maximum volume tolerated. Volumes noted. Maximum fill to 360 mL
d. Recto-anal inhibitory reflex (RAIR) elicited with 50-mL aliquots using rapid balloon inflation with air at 30 mL/s. Volume to first urge and effect on resting pressure noted, maximum 150 mL
e. Participant will receive coaching to evacuate with 60 mL water (one syringe full) in the balloon. Participant will attempt balloon expulsion while the effect on anal pressure is noted
f. The therapist will monitor attempts to relax while pushing and attempting to expel the balloon. Instruct participant to push and breathe, emphasising the need to push from the waist while relaxing the anus. Note propulsive effort. A minimum of 3 and no more than 10 attempts in total, with coaching (therapist observes abdominal and anal activity and advises), or until balloon is expelled (not essential). Therapist will advise on correct pushing technique
g. High-resolution anal manometry (HRaM) can also be used to coach pelvic floor exercises if indicated (e.g. evident perineal descent on pushing)
h. Participants undergoing biofeedback may have rectal hypersensitivity or hyposensitivity. At each interventional visit, these participants will undergo sensitivity training. The goal will be to increase (hypersensitive) or decrease (hyposensitive) tolerated balloon volume by gentle progressive distension or progressively lower volume of air

#### Group 3: INVEST – radio-physiology and stratification (guided treatment)

##### Radio-physiological investigations

Participants allocated to the INVEST arm will undergo standardised investigations (Table 3) prior to stratification of therapy. Routine NHS practice will apply in respect of women between menarche and menopause (that states that radiological examination, if justified, can be carried throughout the cycle until a period is missed). Participants randomised to this group who may potentially be pregnant will have a pregnancy test performed as per routine care. The investigations are shown in Table 3.

**Table 3:** Investigations received by participants in group 3 (INVEST)

a. Anorectal manometry using high-resolution methods [80–82] will be used to determine defined abnormalities of recto-anal pressure gradient (see above for definition of functional defaecation disorder (FDD)) during simulated evacuation [37, 83, 84]. A standard high-resolution anal manometry (HRaM) system will be used with a standard laptop. Calibration, validation and maintenance of the equipment will follow the built-in programme and training manuals provided and will be recorded on the system at each session. Training on the system will be performed and documented in the investigator site file prior to sites commencing treatment
b. Balloon sensory testing using standardised methods [85, 86] (2 mL air per second to maximum 360 mL) will be used to determine volume inflated to first constant sensation, defaecatory desire and maximum tolerated volumes. Rectal hyposensation and hypersensation will be defined in accord to gender-specific normative data on 91 healthy adults [87]. The recto-anal inhibitory reflex will also be elicited by 50 mL rapid inflation (if necessary in 50-mL aliquots up to 150 mL)
c. A fixed-volume (50 mL) water-filled rectal balloon expulsion test [37, 70, 83] will be conducted in the seated position on a commode. Abnormal expulsion is defined as failure to expel with a 1-min effort for men and 1.5 min for women [88]
d. Whole gut transit study will use serial (different shaped) radio-opaque markers over 3 days with single plain radiograph at 120 h [89–91]
e. Fluoroscopic evacuation proctography will use rectal installation of barium porridge to defaecatory desire threshold (or maximum 300 mL) and evacuation on a radiolucent commode [90, 92–95] with preopacification of the small bowel (for enterocoele). Radiation dose, proportion of contrast evacuated and time taken will be recorded, as well as ‘functional’ (i.e. pelvic floor dyssynergia) and ‘structural’ features deemed obstructive to defaecation (e.g. rectocoele, enterocoele and intussusception) [37, 87, 96]. There is a maximum fluoroscopic screening time of 3 min

The results of these investigations will be sent to one of two designated investigators for analysis. A diagnosis of ‘functional defaecation disorder’ (FDD) will be made by assimilation of results from tests in Table 3 by an agreed protocol based on modified ROME III criteria [52]. These results will be reviewed centrally by two expert assessors independently using a secure data-sharing platform (2-week turnaround).

##### Modified Rome III criteria

Note: the updated ROME IV criteria were not available at the time of ethical approval for this study. However, our modified ROME III criteria take into account all investigative modalities (manometry, balloon expulsion and proctography: as Rome IV), and state:

Subjects must have, during repeated attempts to defaecate, evidence of impaired evacuation, based on either:

1. Failed balloon expulsion test, and/or
2. Impaired evacuation on proctography (failure to expel 65% contrast in 150 s),

with or without at least one of the following:

- Inappropriate contraction of the pelvic floor or less than 20% relaxation basal anal pressure on push manoeuvre (manometry) or proctographic evidence of failed puborectalis relaxation or paradoxical contraction; inadequate expulsive forces assessed by manometry or proctography.
- Abnormal pattern of anorectal manometry is defined as inappropriate contraction of pelvic floor or less than 20% relaxation basal anal pressure on push manoeuvre and/or inadequate expulsive forces.
- Based on the results of these investigations, participants diagnosed with FDD will undergo HTBF as above (group-2 interventions). Those without FDD will undergo HT only (group-1 interventions).
- The distribution of stratification factors in group 3, based on modified criteria for FDD [52], are met by approximately 50% of adult patients with CC based on UK data, and thus will be balanced in comparison with patients not receiving investigations. The allocation ratio will be monitored by the Data Monitoring Committee (DMC) (see below).

#### All three groups: concomitant care

Telephone support will be available from the therapist between visits (telephone number given, office hours only). In the instance of new psychological issues being determined during consultation, referral for psychological support will be deferred until after completion of behavioural interventions, except where there is clinical concern regarding the participant’s acute mental state requiring more urgent intervention (see below: withdrawal from treatment criteria).

#### Laxative use

All participants will be asked to discontinue laxative use at the first intervention visit and to refrain from using laxatives throughout the trial, as is standard UK practice in specialist biofeedback services. However, it is inevitable that participants will seek recourse to laxatives and other dietary supplements during the course of the programme. Experience shows that complete prohibition can lead to unreported laxative use, which might confound findings. Although we will strongly discourage ad libitum medication usage and specify a defined breakthrough regimen, we will record co-treatment with sufficient fidelity and integrity to enable use as covariates in analyses using a specific journal for this purpose (within the patient journal: see ‘Study Outcome Measures’). A concomitant medications list, including a shortlist of contributory or confounding medications, will be used to filter on data entry.

### Withdrawal criteria

Individual participants may be withdrawn from treatment in any of the following circumstances:

- Becomes pregnant or intends to become pregnant (only in baseline and intervention phases)
- Subsequently diagnosed with proven cause for secondary constipation, e.g. Parkinson’s disease or bowel obstruction
- Requires new medication with proven effects on bowel function, e.g. opioids
- Develops significant intercurrent illness precluding participation
- Requires surgery or other intervention (other than minor operations) during treatment phase
- Develops acute psychological problem causing safety concern
- Elective withdrawal

All data collected up to the point of withdrawal from treatment will be used in the analyses and data will be collected at further time points unless participants specifically request otherwise. Participants may also request, at any point, no further follow-up visits or data collection.

### Strategies to improve adherence

Participants will be thoroughly counselled from the outset, detailing the study requirements and number of face-to-face treatment visits, the type and nature of all procedures and follow-up data collection visits, including all time commitments and responsibilities when taking part in the study. This will help to improve compliance with the protocol. Additional strategies to improve compliance include a visit window and tolerance of ±1 week and allowance for missing one of four treatment visits, and a follow-up time frame of 6 months without recourse to additional treatments to ensure the primary outcome remains uncontaminated. The participants may then move on to alternative treatments within the NHS routine care pathway or studies 2 or 3 within the CapaCiTY programme. Further strategies include reimbursement for patient travel at study milestones including baseline, 6-month and 12-month follow-up visits.

### Study outcome measures

#### Clinical endpoints

We have adopted a standardised outcome framework throughout the CapaCiTY programme. A set of standard clinical endpoints will be reported in this and the other studies that are part of the programme.

#### Primary clinical endpoint

Self-reported at 6 months after end of intervention:

Response to treatment defined as reduction in the Patient Assessment of Constipation–Quality of Life (PAC-QOL) score (Table 4) of at least 0.4 points [58–60].

**Table 4:** Primary endpoint the Patient Assessment of Constipation–Quality of Life (PAC-QOL) score

The following questions are designed to measure the impact that constipation has had on your daily life over the past 2 weeks. For each question, please check one box.					
	Not at all - 1	A little bit - 2	Moderately - 3	Quite a bit - 4	Extremely - 5
The following questions ask about your symptoms related to constipation. During the past 2 weeks, to what extent or intensity have you…					
1. Felt bloated to the point of bursting?					
2. Felt heavy because of your constipation?					
The next few questions ask about how constipation affects your daily life. During the past 2 weeks, how much of the time have you…					
3. Felt any physical discomfort?					
4. Felt the need to have a bowel movement but not been able to?					
5. Been embarrassed to be with other people?					
6. Been eating less and less because of not being able to have bowel movements?					
The next few questions ask about how constipation affects your daily life. During the past 2 weeks, to what extent or intensity have you…					
7. Had to be careful about what you eat?					
8. Had a decreased appetite?					
9. Been worried about not being able to choose what you eat (for example, at a friend’s house)?					
10. Been embarrassed about staying in the bathroom for so long when you were away from home?					
11. Been embarrassed about having to go to the bathroom so often when you were away from home?					
12. Been worried about having to change your daily routine (for example, traveling, being away from home)?					
The next few questions ask about your feelings related to constipation. During the past 2 weeks, how much of the time have you…					
13. Felt irritable because of your condition?					
14. Been upset by your condition?					
15. Felt obsessed by your condition?					
16. Felt stressed by your condition?					
17. Felt less self-confident because of your condition?					
18. Felt in control of your situation?					
The next questions ask about your feelings related to constipation. During the past 2 weeks, to what extent or intensity have you…					
19. Been worried about not knowing when you are going to be able to have a bowel movement?					
20. Been worried about not being able to have a bowel movement?					
21. Been increasingly bothered by not being able to have a bowel movement?					
The next questions ask about your life with constipation. During the past 2 weeks, how much of the time have you…					
22. Been worried that your condition will get worse?					
23. Felt that your body was not working properly?					
24. Had fewer bowel movements than you would like?					
The next questions ask about your degree of satisfaction related to constipation. During the past 2 weeks, to what extent or intensity have you been…					
25. Satisfied with how often you have a bowel movement?					
26. Satisfied with the regularity of your bowel movements?					
27. Satisfied with the time it takes for food to pass through the intestines?					
28. Satisfied with your treatment?					

#### Secondary clinical endpoints

Self-reported at baseline and at 3, 6 and 12 months after end of intervention:

- Responses to treatment defined as a reduction in PAC-QOL score of at least 0.4 point [58–60]
- Binary responses to treatment defined as either a 1-point (or greater) reduction in PAC-QOL score [58–60]
- PAC-QOL: individual domains and total score (as continuous variables)
- Patient Assessment of Constipation–Symptoms (PAC-SYM) score [61]: individual domains and total score (as continuous variables)
- A 2-week patient diary (for 2 weeks prior to each assessment) to record bowel frequency and whether each evacuation was ‘spontaneous (no use of laxatives) and/or complete’; journal will also capture concurrent medication, health contacts, time away from normal activities (including work) since the patient’s last visit
- Generic quality of life: the EuroQol Health Outcome Measure (EQ-5D-5 L) [62] descriptive system and the EuroQol Visual Analogue Scale (EQ-VAS) [63]
- NHS resource use: interventions, treatment sequelae and other health resource use related to the care of CC
- Patient costs related to constipation and the opportunity cost of time away from normal activities
- Patient Health Questionnaire-9 (PHQ-9) [64, 65]
- Generalised Anxiety Disorder Questionnaire (GAD-7) [66]
- Global study-specific patient satisfaction/improvement score (Visual Analogue Scale: VAS) and whether they would recommend each treatment experienced to other patients
- Potentially modifiable cognitive and behavioural psychological variables shown to predict onset and perpetuation of other functional bowel symptoms: negative perfectionism [67], avoidant and ‘all or nothing’ behaviour subscales of the Behavioural Response to Illness Questionnaire [68] (CC-BRQ), and the Brief Illness Perception Questionnaire (BIPQ (CC)) [69]

A copy of trial outcome measures is available from the trial manager on request.

### Participant timeline

The study flow chart is shown in Fig. 2.

#### Study visits

##### Visit 0: prescreening: eligibility assessment

Patients referred from primary care or identified in secondary or tertiary care clinics will be approached by a suitably trained and delegated local researcher who will screen for basic eligibility by telephone or face-to-face interview (based on patient choice) on the basis of a simplified inclusion/exclusion criteria proforma (i.e. self-reported constipation for more than 6 months, difficulty passing stool and/or infrequent passing of stool, at least two medications tried for relief of symptoms). Patients will be recorded on a screening log and each will be allocated a sequential trial screening number. Patients will be provided with adequate explanation of the aims, methods, anticipated benefits and risks of the relevant interventions and will take away or be posted an invitation letter and a Patient Information Sheet. Patients will be given at least 24 h to consider participation and invited to attend clinic for a more detailed discussion with a suitably trained researcher. The simplified criteria will be used in marketing and promotional materials and will be made available on the study websites with the Participant Information Sheet for patients to self-screen. Those responding to marketing and promotion of the study will be prescreened by the national coordinating centre and referred to their nearest participating trust.

##### Visit 1: screening, consent and baseline assessments

Visit 1 will be conducted face-to-face in clinic. Following a detailed discussion about the trial, eligible and agreeable patients will complete written informed consent. Screening and confirmation of eligibility will be followed by standardised medical and surgical history by interview including previous medication usage. Clinical examination findings (carried forward if performed previously within last 3 months) and standardised examination of the perineum/anus/rectum/vagina will be performed.

Baseline assessments include several key validated assessments that profile patients for important characteristics informing disease pathophysiology and important potential predictors of treatment response. This includes the constipation and irritable bowel syndrome modules of the Rome III questionnaire [70], the Cleveland Clinic Constipation Score Questionnaire [71], brief chronic pain, autonomic symptoms [72] and joint hypermobility [73] assessments and St. Mark’s Faecal Incontinence Score [74]. All were selected on the basis of trade-off between adequate detail and brevity. These instruments have been collated into a single booklet with design and presentation optimised by patient representatives. Participants will complete the questionnaire booklet (containing baseline, primary and secondary outcomes listed above), and be given a baseline 2-week patient diary and journal. All participants will be asked to complete the 2-week patient diary prior to INVEST, HT or HTBF interventions and prior to stopping laxatives.

Urinary pregnancy testing will be made available to women of child-bearing potential at eligibility assessment and advice will be given to all women regarding the need to prevent pregnancy during the study intervention period. Randomisation to HT-, HTBF- or INVEST-guided intervention will be completed at the end of visit 1.

##### Visits 2–5: interventions

Participants randomised to treatment without INVEST will undergo four sessions of standardised therapy (HT or HTBF) (Tables 1 and 2). Those randomised to INVEST will have additional radio-physiological investigations (Table 3) prior to being stratified to receive either HT or HTBF. A review of all related AEs and changes in confounding medications will be conducted at each intervention visit.

##### Visits 6–8: follow-up outcome assessments (3, 6 and 12 months)

The standardised outcome questionnaires booklet will be completed and the 2-week Bowel Diary and Patient Journal completed and returned, including a review of all related AEs and changes in confounding medications. In order to maximise completeness of data collected, the 3- and 6-month follow-up visits will be conducted face to face wherever possible. Follow-up at 12 months can, however, be conducted by telephone. The 6-month time point at which the primary outcome is recorded will coincide with the end of a protocol-imposed ‘quarantine’ period during which a participant’s response to therapy will remain unconfounded by treatment progression.

### Sample size for the trial

The sample size has been calculated using the primary clinical outcome change in PAC-QOL score. A 0.4-point (10% of scale) reduction in PAC-QOL score [75–77] with a variance estimate conservatively set at a standard deviation (SD) = 1 will be considered clinically relevant.

To detect a mean change of 0.4 in PAC-QOL score (SD = 1) with 90% power and 5% significance level, 132 per arm or 264 participants in total will be required for the comparison of HT and HTBF (No-INVEST arm).

For the secondary comparison of INVEST versus No-INVEST a reduction of 0.4 points (SD = 1) will also be considered clinically meaningful. To detect an effect size of 0.4 with 90% power and at 5% significance level requires 90 participants in the INVEST arm assuming 264 participants have been recruited to the No-INVEST arm.

Allowing for a 10% loss to follow-up, a sample size of 147 is needed in the HT and HTBF arms and 100 in the INVEST arm. A total sample size of 394 patients across the three arms is required.

### Recruitment

All recruiting sites have been chosen based on feasibility assessments and ability to recruit the required number of participants during the recruitment time frame. An assessment of competing studies, research experience and required resources to conduct the study was also undertaken to ensure that targets can be achieved. The recruitment rates at each site will be monitored throughout the course of the study and barriers to recruitment identified and addressed as they arise. Reserve sites have been identified to address any shortfalls in recruitment. If a larger than anticipated dropout rate occurs, it will be possible to recruit further patients above that suggested in each arm to maintain study power.

### Assignment for interventions

#### Sequence generation and allocation

Randomisation sequence will be computer-generated. Randomisation will take place after consent, eligibility and baseline assessments. We will use a secure, online, access-controlled randomisation system managed by the UKCRC registered, Barts and the London, Pragmatic Clinical Trials Unit (PCTU).

#### Stratification for therapy

As for previous studies in this area we expect to recruit very few men. To ensure balanced randomisation within gender, and secondarily within centres, randomisation will be stratified by gender and then women by centre.

#### Allocation concealment mechanism

The randomisation system is user-access-controlled to maintain allocation concealment from blinded individuals.

#### Blinding

Patients and clinicians are necessarily aware of both INVEST and treatment allocations after randomisation. The primary outcome and several other outcomes are self-reported. All those involved in developing the statistical analysis plan will be blinded to allocation status until the plan is signed off, and no analyses of outcomes will be undertaken until that is done. To minimise observer bias, a blinded researcher will collect outcome data. If a blinded researcher is unavailable, the primary outcome questionnaire will be completed by the participant without assistance and secured in an envelope.

Statisticians performing the analysis will remain blinded.

## Data collection, management and analysis

### Data collection

Data collection methods consist of a mixture of routine clinical data collected by investigators and patient-reported outcomes. Validated questionnaires have been chosen for the majority of the primary and secondary outcome measures, with the exception of the Patient Bowel Diary and Health Utilisation Journal. All participants will be trained in completing the questionnaires at baseline, prior to randomisation and will be provided with a standardised guideline for completing questionnaires to minimise errors or missing data. When available, a blinded outcome assessor will check to verify and confirm questionnaire completion. In order to minimise missing data, follow-up visits up to 6 months post treatment will be conducted face to face. Thereafter, data can be collected over the telephone or via mail). All data will be collected on intention-to-treat principles regardless of protocol excursions and deviations or withdrawal from trial treatments. At least three attempts via two different methods (e.g. telephone and letter), will be made by research staff to make contact and collect follow-up data, after which the participant may be considered lost to follow-up. Participants may be lost to follow-up in the following circumstances:

- After at least three failed attempts by research staff to make contact via two different methods (e.g. telephone and letter)
- Participant does not wish to participate in follow-up data collection
- Death or significant incapacity making follow-up data collection impossible

A copy of all Data Collection Forms is available from the trial manager on request

### Data management

Each recruiting site will be required to keep accurate and verifiable source notes in the medical record relevant to each study participant’s inclusion and continued participation in the study.

Data will be collected, transferred and stored in accordance with Good Clinical Practice (GCP) guidelines and data protection requirements. The PCTU SOPs and study data management plan and SOP will define the exact process of data collection, transfer, storage and quality control of study data.

A secure online trial database will be provided by the PCTU to enable remote data entry at sites where this is feasible. This database will provide built-in data validation range checks. Additional quality control will be performed by the central study team on the study data prior to review by the DMC and statistician. These checks will consist of:

- Repeating data entry (into a separate data table/database provided by the data manager) for a random sample, 10% of the baseline and final outcome data and comparing it with the main database. Discrepancies will be checked against the Case Report Form (CRF) to ensure data entry errors on validation are not counted and only those errors that occurred within the main database will be counted. These errors will be cleaned and corrected
- Values for each variable will be sorted by the statistician, and those at the extremes will be checked to ensure that they are either correct or within the expected range

Error rates of 1% for primary outcomes and 3% for the remaining data will need to be achieved. The error rate (*r*) will be calculated by comparing the number of errors (*e*) with the total number of CRF fields (*t*): *r* (%) = (*e*/*t*) × 100. If the prespecified error rates are not achieved, data checking and cleaning will be repeated (in additional random 10% lots) and continue until the error rate is met. Missing data will be handled according to the statistical analysis plan. In addition on-site monitoring will enable source document verification of records and will follow the PCTU trial monitoring SOPs and the trial monitoring plan.

All patient-identifiable data, such as Consent Forms, screening and identification logs will be stored in the investigator site files in secure, locked cabinets and offices, accessible only to delegated members of the study team. Secure methods of data transfer will be used to return CRFs to the coordinating site for centralised data entry, monitoring, quality control and in compliance with GCP. A copy of the CRF is held at the site in accordance with GCP.

### Statistical methods

#### Clinical outcomes analysis

The primary outcome will be analysed on intention-to-treat at the 6-month time point (prior to progressing to further condition-specific care). The average reduction in PAC-QOL score will be analysed using linear mixed-model regression with a random effect for centre and fixed effects for intervention, gender, baseline PAC-QOL and breakthrough medication.

Secondary outcomes will be analysed at 6-months and at additional time points (3 and 12 months from end of treatment) including the percentage of patients achieving a 1-point reduction. Outcomes will take the form of count (change in number of symptom episodes), ordinal (patient’s global impression of success) and continuous (questionnaire scores) data. Regression models, with a random effect for centre, appropriate to the outcome data types will be fitted to estimate the treatment effect, adjusting for baseline values (when appropriate), gender, and breakthrough medication use as a potential confounder.

Predictive modelling using baseline characteristics: measures of chronic pain, autonomic symptoms, joint hypermobility, cognitive, behavioural and mood variables share the common hypothesis that they are detrimental to the success of all treatments, i.e. they perpetuate illness in spite of therapy. Appropriate regression models will be developed to determine the influence of these pretreatment characteristics on the success of treatments.

Analysis will be performed using proprietary software (Stata, Stata Corp., College Station, TX, USA) by the appointed study statistician. *P* < 0.05 will be taken to indicate statistical significance. No analyses will be conducted until a statistical analysis plan (SAP) has been written and reviewed by an independent statistician. The SAP will be approved by the senior statistician and chief investigator. Multiple imputation will be used to address missing values. Subgroup analyses will be performed for selected baseline characteristics.

#### Health economic outcome analysis

The pragmatic multicentre trial design reflects real-world clinical practice, thus cost and outcome profiles are likely to reflect routine care in NHS settings. Individual patient data collected within the trial included NHS treatment and personal costs as well as health status estimated as quality-adjusted life years (QALYs). Cost-effectiveness analysis will capture the effect of treatment as changes in cost and QALYs. The base case analysis will proceed using multiple imputation of patient data to manage missing values, following good practice guidance [78, 79]. Imputed datasets will be analysed independently and the estimates obtained will be pooled to generate mean and variance estimates of costs and QALYs using Rubin’s rule [75]. Each analysis will use a bivariate regression modelling approach to capture the correlation of costs and outcomes as well as covariates within each model. Models will be bootstrapped with 5000 replicates, using Stata. Supportive sensitivity analyses, including complete case analysis, will be used to explore the impact of imputation and missing data.

Cost-effectiveness models that extrapolate beyond 3–6 months’ duration are problematic in adult constipation, as outcomes are contingent upon subsequent care received and the underlying disease process. However, the programme of work packages provides a unique opportunity to construct probabilistic models exploring optimal pathways from effectiveness and cost-effectiveness perspectives. Since patients will (within the CapaCiTY programme) be followed along a pathway that includes a series of steps of care, it will be possible to construct costs and outcomes for a range of patient pathways providing comparative longer-term cost-effectiveness estimates. For example, it will be possible to ask whether INVEST or No-INVEST-led first-line care leads to lower overall costs or improved outcomes. For the overall CapaCiTY programme of studies, patient-level data from recruitment through the various work packages will be used to construct pragmatic, probabilistic models to explore optimal pathways from effectiveness and cost-effectiveness perspectives.

Since EQ-5D and EQ-VAS typically have SDs of 30% of scale: a 10% difference deemed clinically significant can be detected with the large sample sizes proposed within the trial. Adjustment for time preference will be at the socially accepted rate for cost-effectiveness analyses (currently 3.5% for costs and benefits). Patient use of resources and EQ-5D values will be used to estimate the cost-effectiveness of the three comparisons being studied. A base case analysis will estimate within trial incremental cost/quality-adjusted life years (QALYs) gained from an NHS perspective, with missing values imputed and with adjustment for trial covariates. A range of supportive sensitivity analyses will include a complete case analysis, effect of covariate adjustment and analysis perspective.

#### Nested qualitative study: patient and health professional experience

Face-to-face, semistructured interviews will be conducted involving a diverse sample of patients and professionals purposefully selected at various time points throughout the study:

1. Before starting to gauge expectations
2. During treatment to explore fidelity and ease of adherence
3. Immediately after, and up to 2 years after, intervention, to explore perspectives on the intervention and longevity of any benefit and pattern of response over time

Participants for interview will be selected from a sampling grid of potential interviewees from all three arms, both improved and not improved by intervention, to reflect a range of ages, geographical locations and, where possible, other pertinent attributes such as ethnicity and gender. Approximately 50 interviews (or until apparent data saturation) will be conducted with participants. We will also interview 10 professionals delivering the intervention, to determine comparative ease of delivery of the two interventions.

Separate informed consent will be taken for interviews (see Additional file 2 for Consent Form). Interviews will be conducted throughout to capture relatively early and later experiences and perceptions of the interventions. A topic guide for the interviews, informed by the existing literature and our patient advisors, has been developed.

#### Qualitative analysis

Interviews will be digitally recorded, anonymised, transcribed verbatim and analysed using a pragmatic thematic analysis and NVivo8 software (QSR International Ltd., Warrington, UK) for data management. Data analysis will be developed as outlined by Fereday and Muir-Cochrane [78] in the first instance by mapping key concepts derived from the transcripts (‘charting’) and extracting emergent themes from the transcripts. A second researcher will conduct independent analyses and then compare and refine resulting codes and themes in discussion. Emergent themes, together with captured observational data, will form the basis of analytical interpretation.

### Monitoring and audit

The PCTU quality assurance manager will conduct a study risk assessment in collaboration with the CI. Based on the risk assessment, an appropriate study monitoring and auditing plan will be produced according to PCTU SOPs. This monitoring plan will be discussed and authorised by the sponsor before implementation. Any changes to the monitoring plan must be agreed by the PCTU quality assurance manager and the sponsor. Audits may be conducted by the sponsor or funder representative. The study may be identified for audit via the risk assessment process, investigator or department request, allegation of research misconduct or fraud or a suspected breach of regulations or selected at random.

### Trial committees

The trial and the whole CapaCiTY programme will be overseen by a Programme Steering Committee (PSC). The role of the PSC is to provide overall supervision of the study on behalf of the sponsor and funder to ensure that the study is conducted in accordance with the principles of GCP and relevant regulations.

The responsibilities of the PSC will include: ensuring that views of users and carers are taken into consideration; advising on the trial protocol; advising on changes in the protocol based on considerations of feasibility and practicability; assisting in resolving problems brought to it by the Programme Management Group (PMG); monitoring the progress of the trial and adherence to protocol and milestones; considering new information of relevance from other sources; considering and acting on the recommendations of the Data Monitoring and Ethics Committee (DMEC), sponsor and/or Research Ethics Committee (REC); reviewing trial reports and papers for publication.

The PMG will meet monthly initially during study set-up and then every 2 months. The PMG will be responsible for day-to-day project delivery across participating centres, and will report to the PSC.

The DMEC will meet at least 4 weeks prior to the PSC to enable recommendations to be fed forward. The DMEC will comprise an independent chair an independent statistician and a clinician.

A Constipation Research Advisory Group (CRAG) will comprise eight patients and two lay members. This group will have geographical diversity and a disease-appropriate demographic (eight women, two men). The CRAG will be involved in review of participant information sheets, booklets, diaries and advertising/marketing materials, project management by representation on the PSC, parallel qualitative analysis, dissemination of results and lay summaries, and presentations at local research events.

There are no plans for interim analysis as this is a low-risk study.

### Harms

The risks arising from participation are considered very low. The interventions proposed are those already offered to patients in specialist centres throughout the UK and internationally. The only difference conferred by participation is that these interventions will be randomly allocated and more carefully assessed. All interventions are safe. For instance, the only invasive tests (INVEST) have been performed daily in most specialist centres for up to 30 years without any recorded complication (experience of the lead centre is more than 122,000 patients). A small ionising radiation dose is required for two tests. All devices used in the study are CE-marked and will be used in accordance with manufacturer’s instructions and intended uses.

A number of questionnaires contain personal questions about bowel problems and the effect of these on quality of life and psycho-behavioural functioning; however, all have been used in studies of similar patients previously.

The benefits of participation are that patients will receive a very high standard of monitored care as a consequence of the detailed protocol. In some instances (geographically), patients may receive interventions for which they did not previously have access.

There are minimal safety considerations attributable to the interventions. Patients allocated to INVEST-guided therapy will undergo two radiological procedures (whole gut transit study and evacuation proctography) using ionising radiation as outlined above. The combined dose of these procedures (approximately 1.2 mSv) is equivalent to less than 7 months’ annual background radiation dose from living in the UK. Further, these investigations would be carried out in routine clinical practice in many centres for patients at the same point as recruitment to this study. There are rare reports of local irritation or discomfort subsequent to manometry and very rare reports of rectal perforation after manometry [79], but only in the presence of prior radiotherapy and surgery.

As no medicinal products are being administered as part of the trial and all trial interventions are as per the standard care provided within the NHS for chronic constipation, unrelated AEs will not be recorded on the CRF. Causality will be at the discretion of the health care provider. Serious adverse events (SAEs) will be recorded on the CRF and in the medical notes to enable assessment and reporting in line with sponsor and regulatory requirements.

Trial participants will be advised to seek medical support from their family physician for any unrelated signs, symptoms or disease or aggravation of underlying symptoms.

SAEs that are considered to be ‘related’ and ‘unexpected’ are to be reported to the chief investigator and sponsor within 24 h of the site learning of the event and by the coordinating team to the REC within 15 days in line with the required time frame.

The following SAEs are expected to occur rarely in this patient population and will not be reported:

- Hospital admission for exacerbation of constipation symptoms including impaction
- Hospital admission for unrelated elective surgical procedures or accidental injury

In the event that participants suffer harm during the research study, and this is due to someone’s negligence, then participants may have grounds for legal action against the sponsor Queen Mary University of London. Insurance and indemnity is provided by the sponsor to cover such claims.

### Criteria for discontinuation

The interventions proposed are well-established in current clinical practice and have minimal safety concerns. There are no defined criteria for discontinuation. Additionally, if the DMEC, PSC, REC or sponsor determine that it is within the best interests of the participants or trial to terminate the study, written notification will be given to the chief investigator. This may be due to, but not limited to, safety concerns, proof of effectiveness or serious and persistence noncompliance/serious breaches. If the study is terminated, participants will be returned to the NHS for normal follow-up and routine care.

### Ethical issues

#### Confidentiality

Information related to participants will be kept confidential and managed in accordance with the Data Protection Act, NHS Caldecott Principles, The Research Governance Framework for Health and Social Care, and the conditions of REC approval. All CRFs will be pseudonymised. The participant’s GP will be informed of their participation in the study.

### Access to data

All authors of the final trial report will have full access to data with no restrictions. Trial records will be archived for a period of 20 years.

### Post-trial care

Participants who do not achieve what they consider to be adequate relief of symptoms following trial completion will be referred back to the NHS pathway of care and considered by the local clinical team for further care. They may be offered biofeedback if this has not already performed within the study or be eligible to progress to other work packages in the CapaCiTY programme (rectal irrigation and consideration for laparoscopic ventral mesh rectopexy surgery). Alternatively, they may be offered these or other treatments outside the study.

### Dissemination

Results of the trial will be prepared for presentation at relevant scientific meetings and submitted to a leading scientific journal for publication. All protocol authors meeting journal criteria will be eligible as authors of these works and no professional writers will be utilised. With our patient representatives we will prepare a lay summary for the public. The results will also feed into the development of a care pathway for chronic constipation as part of the wider CapaCiTY programme grant. We have no plans to make the dataset publically available.

## Discussion

This trial has the potential to answer some of the major outstanding questions in the management of the common problem of chronic constipation which is resistant to primary care interventions. It should enable decisions about whether costly invasive tests are warranted prior to specialist management and whether computer-assisted biofeedback confers additional benefit to well-managed conservative specialist advice alone.

### Trial registration

The trial is registered on a publically accessible registry: ISRCTN11791740 (date of registration 16 July 2015); http://www.isrctn.com/ISRCTN11791740.

### Trial status

The trial commenced recruitment in March 2015 and will take 30 months to recruit 394 patients. Recruitment milestones will be closely monitored.

## Contacts

For scientific enquiries: CI: Professor Charles Knowles: c.h.knowles@qmul.ac.uk.

For public and administrative enquiries: trial manager: Natasha Stevens: n.stevens@qmul.ac.uk.

## Additional files

Additional file 1: SPIRIT Checklist. (DOC 121 kb)

Additional file 2: Study Consent Form. (DOCX 47 kb)

Additional file 3: Consent form for interviews. (DOCX 48 kb)

## Abbreviations

AE: Adverse event
BIPQ: Brief Illness Perception Questionnaire
CC: Chronic constipation
CRF: Case Report Form
DMEC: Data Monitoring and Ethics Committee
EQ-5D: EuroQol Health Outcome Measure
EQ-VAS: EuroQol Visual Analogue Scale
FDD: Functional defaecation disorder
GAD-7: Generalised Anxiety Disorder Questionnaire
HT: Habit training
HTBF: Habit training plus computer-assisted direct visual biofeedback
INVEST: Standard panel of radio-physiological tests of colonic and anorectal function
PAC-QOL: Patient Assessment of Constipation Quality of Life Questionnaire
PAC-SYM: Patient Assessment of Constipation Symptoms Questionnaire
PHQ-9: Patient Health Questionnaire-9
PMG: Programme Management Group
PSC: Programme Steering Committee
QOL: Quality of life
RAIR: Recto-anal inhibitory reflex
RCT: Randomised controlled trial
REC: Research Ethics Committee
SAE: Serious adverse event
SD: Standard deviation
VAS: Visual Analogue Scale
