# Supplementary material for: Habit training versus habit training with direct visual biofeedback in adults with chronic constipation: study protocol for a randomised controlled trial
Source: Trials. 2017 Mar 24;18:139. doi: 10.1186/s13063-017-1880-0 (PMC5366116; doi:10.1186/s13063-017-1880-0)
Supplement: Supplementary file 2 — Study Consent Form. (DOCX 47 kb) [file 13063_2017_1880_MOESM2_ESM.docx]

**Additional file 2: STUDY CONSENT FORM**

Title of Project: **Chronic Constipation Treatment Pathway, Study 01**

Name of Researcher: **<insert local Principal investigator name and title>**

**<Local investigator contact details>**

| **Study ID:** |  |  | **-** |  |  |  | **-** |  |  |  |  |
| --- | --- | --- | --- | --- | --- | --- | --- | --- | --- | --- | --- |

|  | **Place initials in each box** |
| --- | --- |
| 1. I confirm that I have read and understand the Patient Information Sheet dated **[DATE]** (version **[VERSION NUMBER]**) for the above study. I have had the opportunity to consider the information, ask questions and have had these answered satisfactorily. |  |
| 1. I understand that my participation is voluntary and that I am free to withdraw at any time without giving any reason, without my medical care or legal rights being affected. |  |
| 1. I understand that relevant sections of my medical notes and data collected during the study may be looked at by individuals from Queen Mary, University of London, from regulatory authorities or from the NHS Trust, where it is relevant to my taking part in this research. I give permission for these individuals to have access to my records. |  |
| 1. I agree to undergo GI Physiological tests including tests using X-rays. |  |
| 1. I agree for my contact details and relevant identifiable data to be passed to researchers from Queen Mary University of London or Kings College London. |  |
| 1. I agree to my GP being informed of my participation in the study. |  |
| 1. I agree to my data being used in future related research |  |
| 1. I agree to take part in the above study. |  |

|  |  |  |  |  |
| --- | --- | --- | --- | --- |
| *Print Name of Participant* |  | *Date* |  | *Participant’s Signature* |
|  |  |  |  |  |
| *Print Name of person taking consent* |  | *Date* |  | *Signature of person taking consent* |
