# Supplementary material for: Habit training versus habit training with direct visual biofeedback in adults with chronic constipation: study protocol for a randomised controlled trial
Source: Trials. 2017 Mar 24;18:139. doi: 10.1186/s13063-017-1880-0 (PMC5366116; doi:10.1186/s13063-017-1880-0)
Supplement: Supplementary file 3 — Consent form for interviews. (DOCX 48 kb) [file 13063_2017_1880_MOESM3_ESM.docx]

**INTERVIEW CONSENT FORM**

Title of Project: **Chronic Constipation Treatment Pathway, Study 01**

Name of Researcher: **<insert local Principal investigator name and title>**

**<Local investigator contact details>**

| **Study ID:** |  |  | **-** |  |  |  | **-** |  |  |  |  |
| --- | --- | --- | --- | --- | --- | --- | --- | --- | --- | --- | --- |

|  | **Place initials in each box**  **(If researcher is taking consent on behalf of patient via remote recording, place researcher initials in each box)** |
| --- | --- |
| 1. I confirm that I have read and understand the Information Sheet dated **[DATE]** (version **[VERSION NUMBER]**) for the above study. I have had the opportunity to consider the information, ask questions and have had these answered satisfactorily. |  |
| 1. I understand that my participation is voluntary and that I am free to withdraw at any time without giving any reason, without my medical care or legal rights being affected. |  |
| 1. I agree to take part in a 60 minute interview and I understand that this will be audio taped and transcribed. |  |
| 1. I agree to take part in the above study. |  |

|  |  |  |  |  |
| --- | --- | --- | --- | --- |
| *Print Name of Participant* |  | *Date* |  | *Participant’s Signature* |
|  |  |  |  |  |
| *Print Name of person taking consent* |  | *Date* |  | *Signature of person taking consent* |

Was the interview: face-to-face *or* remote (via telephone/Skype?)

If consent was taken remotely, please confirm that this was recorded: Yes/ No (please circle)

One copy for participant, one copy for researcher (kept on file)
